# Immune response in fluid therapy with crystalloids of different ratios or colloid for rats in haemorrhagic shock

**DOI:** 10.1038/s41598-020-65063-4

**Published:** 2020-05-15

**Authors:** Eun-Hye Seo, Hyun Jun Park, Li-Yun Piao, Ji Yeon Lee, Chung-Sik Oh, Seong-Hyop Kim

**Affiliations:** 1 BK21 Plus, Department of Cellular and Molecular Medicine, Konkuk University School of Medicine, Seoul, Korea; 2 Department of Infection and Immunology, Konkuk University School of Medicine, Seoul, Korea; 3 Department of Anaesthesiology and Pain medicine, Konkuk University Medical Center, Konkuk University School of Medicine, Seoul, Korea; 4 Department of Medicine, Institute of Biomedical Science and Technology, Konkuk University School of Medicine, Seoul, Korea

**Keywords:** Trauma, Trauma, Experimental models of disease, Experimental models of disease

## Abstract

This study investigated the association between different ratios of balanced salt based-crystalloid (PLASMA SOLUTION-A [CJ HealthCare, Seoul, Korea]) (the ratios of crystalloid for blood loss, 1:1, 1:2 and 1:3) or balanced salt-based colloid (VOLULYTE 6% [Fresenius Kabi, Germany]) (the ratio of colloid for blood loss, 1:1) to restore blood loss and immune response in rats with haemorrhagic shock. About 50% of total estimated blood volume was removed after anaesthesia. The fluid was administered for resuscitation after exsanguination, according to the type of fluid and the ratios of exsanguinated volume and fluid volume for resuscitation. After sacrifice, expression of immune cells in blood and tissues was evaluated. Histological analyses and syndecan-1 immunohistochemistry assays were performed on tissues. Endothelial damage according to syndecan-1 and cytokine levels in blood was also assessed. Fluid resuscitation with same, two-fold, or three-fold volumes of crystalloid, or same volume of colloid, to treat haemorrhagic shock in rats resulted in a similar increase in blood pressure. The expression of neutrophils in blood decreased significantly after colloid administration, compared to before exsanguination. Syndecan-1 expression increased after exsanguination and fluid resuscitation in all groups, without any significant difference. In conclusion, same volume of balanced salt-based crystalloid for blood loss was enough to restore BP at the choice of fluid for the management of haemorrhagic shock in the rats, compared with different ratios of crystalloid or same volume of colloid, on the aspect of immune response.

## Introduction

Haemorrhagic shock is one of hypovolemic shock and defined as the condition of life-threatening blood loss with decreased blood flow to the tissues, resulting in inadequate tissue function and cellular injury. The primary management is fluid therapy to replace the lost blood and control of the haemorrhagic source. The first choice of replacement fluid is crystalloid or synthetic colloid. Hydroxyethyl starch (HES) in synthetic colloid is used to maintain intravascular volume but is associated with side effects, such as hepatic dysfunction, renal dysfunction, coagulopathy and so on^1^. Recently, balanced salt-based solution, regardless of crystalloid or colloid, for the management of blood loss has been favored because normal saline-based solution is associated with side effects, such as hyperchloremic metabolic acidosis and coagulopathy^2^. Crystalloid is administered at the same to three- or four-fold volumes of blood loss because it quickly leaks from the intravascular space^3^. Alternatively, synthetic colloid restores blood volume at a 1:1 ratio^4^. However, recent trials have demonstrated that less crystalloid is sufficient^5–7^. The trials have also showed that organ function was different, according to the type of fluid and amount of fluid, even though restoration of haemodynamic parameters was similar. Therefore, we hypothesized that the immune response might be dependent on the type of fluid such as crystalloid versus colloid, and the amount of fluid.

We investigated the association between balanced salt-based crystalloid (PLASMA SOLUTION-A [CJ HealthCare, Seoul, Korea]) with different ratios or balanced salt-based colloid (VOLULYTE 6% [Fresenius Kabi, Germany]) to restore blood loss, and the immune response in rats with haemorrhagic shock.

## Materials and Methods

After approval by the Institutional Animal Care and Use Committee (IACUC) of the Konkuk University (approval number: KU2017094) on September 04 2017, .all experiments, following the IACUC guidelines, were conducted at the Konkuk University Laboratory Animal Research Center. All procedures from *Experiment design* to *Statistics* in Materials and Methods were standardized by our institutional protocols, and followed our previous studies^8–10^. The data used to support the findings of the study are available from the corresponding author (Seong-Hyop Kim, yshkim75@daum.net) upon request.

### Experiment design

Male Sprague–Dawley rats with age of 6–8 weeks and body weight of 200 g were purchased from Orient Bio Inc. (Seongnam, Korea). The animal experiments were carried out based on the National Institutes of Health guidelines for care. All animals were acclimated and handled for 7 days before starting the experiments.

As described in previous studies^9,10^, anaesthesia was performed. Anaesthesia was induced by intraperitoneal injection of 20 µg/g xylazine (ROMPUN, Bayer Korea Ltd., Seoul, Korea) and checked by pinching the hind foot. Tracheal intubation was performed on a surgery platform. A heating pad with 37.5 °C was placed between the surgery platform and the table to maintain body temperature during the surgery. Room temperature was also maintained at 25 °C to prevent hypothermia. After placing a rat in a supine position and fastening it to the platform with tape, the tongue was pulled out with forceps. A 16 G catheter 4.50 cm (BD, Franklin Lakes, NJ, USA) was inserted through the larynx to the bronchus. The correct position of the catheter for intubation was confirmed by checking for symmetrical chest expansion. A ventilator was connected to the catheter for intubation. The ventilator settings were as follows: fraction of inspired oxygen, 0.5; inspiratory flow rate, 170 mL/min; tidal volume (TV), 1.70 mL, and respiratory rate (RR), 50 breaths/min. Anaesthesia was maintained with isoflurane (JW Pharmaceutical, Seoul, Korea) 3 volume% via the intubation catheter. Actual TV with RR was monitored during mechanical ventilation. A tail cuff was applied for non-invasive systemic blood pressure (BP) monitoring (AD Instruments, Sydney, Australia) after setting the ventilator and administering the isoflurane. A right inguinal skin incision was made, and subcutaneous tissue around the right femoral artery and vein was carefully dissected to expose them. After confirmation of them, a 29 G catheter was placed in the right femoral artery to induce haemorrhagic shock and the femoral vein to resuscitate with fluid, respectively. For no-touch at the line for fluid administration, the left femoral artery, instead of the right femoral artery, was used for blood samples. The left inguinal incision was made and subcutaneous tissue around the left femoral artery was carefully dissected to exposure it. After confirmation of it, a 29 G catheter was placed in the left femoral artery to collect the blood samples. The catheters were fixed with a 6-0 silk surgical suture. Haemorrhagic shock was induced by exsanguination. Total estimated blood volume (TEBV) was defined as 0.064 mL/g based on a previous study^6^ and 50% of TEBV was removed using a 5 mL syringe. Fluid was administered to resuscitate through the left femoral vein using a syringe pump (Harvard Apparatus, Holliston, MA, USA) at 60 min after exsanguination. According to the type of fluid (PLASAM SOLUTION-A [CJ HealthCare, Seoul, Korea] as the crystalloid and VOLULYTE 6% [Fresenius Kabi, Germany] as the colloid) and the ratios of the exsanguinated volume and fluid volume for resuscitation (1:1 vs. 1:2 vs. 1:3), the experimental animals were divided into four groups: the crystalloid-1 group, 1:1 crystalloid; crystalloid-2 group, 1:2 crystalloid; crystalloid-3 group, 1:3 crystalloid; and colloid group, 1:1 colloid. The compositions of the fluids were summarized in Table 1. The different amount of the fluid, determined by the group, was administered for same duration, 10 min. BP was measured before (T0), 10 (T1), 20 (T2), and 30 min after exsanguination (T3), and 10 (T4), 20 (T5), and 30 min after resuscitation (T6). The experimental animals were sacrificed 60 min after fluid administration.

**Table 1:** Composition of PLASMA SOLUTION-A as the crystalloid and VOLULYTE 6% as the colloid in the study.

	PLASMA SOLUTION-A	VOLULYTE 6%
Hydroxyethyl starch (g/L)	—	60
pH	6.5-8.0	5.7-6.5
Osmolarity (mOsmol/L)	295	286.5
Sodium (Na^+^) (mEq/L)	140	137
Potassium (K^+^) (mEq/L)	5.0	4.0
Calcium (Ca^2+^) (mEq/L)	—	—
Magnesium (Mg^2+^) (mEq/L)	3.0	1.5
Chloride (Cl^−^) (mEq/L)	98	110
Acetate (mEq/L)	27	34
Gluconate (mEq/L)	23	—

The expression of immune cells in the blood and the tissues was evaluated among the groups. Histopathological analyses and syndecan-1 immunohistochemical assays of the heart, lungs, and kidneys were performed to check the impact of immune response on tissue injury. Endothelial damage was assessed based on blood syndecan-1 and cytokine levels.

### Isolation of cells from blood, heart, lungs, and kidneys

Blood samples of 0.25 mL were taken from the left femoral artery to check immune cells and endothelial damage according to syndecan-1 and cytokine levels at T0, T3, and T6. The blood was collected in tubes precoated with ethylenediaminetetraacetic acid (EDTA). Peripheral blood mononuclear cells (PBMCs) were isolated from the blood using density-gradient centrifugation over a Biocoll gradient solution (Biochrom Ltd., Cambridge, UK). After sacrifice, the heart, lungs, and kidneys were extracted and minced into 1 mm^3^ pieces on ice. After washing, the tissues were digested with 1 mg/mL collagenase type 1 (Sigma-Aldrich, St. Louis, MO, USA) in 5 mL phosphate-buffered saline (PBS) at 37 °C for 60 min. After incubation, mononuclear cells in the digested solution were filtered through a 70 µm cell strainer (SPL Life Science Co., Pocheon, Korea).

### Flow cytometry

Neutrophils, cluster of differentiation (CD)4^+^ T cells, CD8^+^ T cells, and CD4^+^CD25^+^ T cells were evaluated in isolated blood and tissues. PBMCs and cells from each tissue were washed with fluorescence activated cell sorter buffer (1% bovine serum albumin and 0.01% sodium azide in PBS). Then they were stained with retinitis pigmentosa-1, CD11b, CD4, CD8, and CD25 antibodies to detect immune cells. Staining was performed for 30 min in the dark at room temperature, and the samples were analysed on a flow cytometer. The data were analysed using FLOWJO software (BD).

### Histopathological analyses

As described in previous studies^8–10^, histopathological analyses were performed. Tissues were fixed overnight at 25 °C in 4% paraformaldehyde solution (Biosesang, Seongnam, Korea) and embedded in paraffin blocks. They were cut into slices 4 µm thick using a microtome and stained with haematoxylin (Vector Laboratories, Burlingame, CA, USA) and eosin (Sigma-Aldrich). The sliced tissues were examined by light microscopy. A heart injury score was determined in sections containing right and left ventricles using a semi-quantitative scale from 0 to 4, as follows: 0, no injury; 1, isolated myocyte injury; 2, one focal area of injury; 3, two or more areas of injury; and 4, diffuse areas of damage making up >50% of the myocardium. Lung injury was scored as follows: 0, no injury; 1, alveolar congestion; 2, with haemorrhage; 3, infiltration or aggregation of neutrophils in the airspace or vessel wall; and 4, thickening of the alveolar wall/hyaline membrane formation. A kidney injury score was defined by the degree of tubular cell damage from 0 to 4: 0, no damage; 1, unicellular or patchy isolated necrosis; 2, tubular necrosis <25%; 3, tubular necrosis of 25–50%; and 4, >50% tubular necrosis with infarcted tissue. Images were taken through a microscope (Nikon, Tokyo, Japan) and tissue injury was quantified. For the area of tubular necrosis in kidney injury score, ImageJ software (National Institutes of Health, Bethesda, MD, USA) was used.

### Syndecan-1 immunohistochemical assay

The slides were deparaffinized and rehydrated before staining. To avoid nonspecific antibody binding, the slices were incubated in blocking solution (Vector Laboratories) for 1 h and reacted with anti-rat syndecan-1 antibody (Santa Cruz Biotechnology, Dallas, TX, USA) at a 1:100 dilution overnight at 4 °C. The slides were washed in PBS, and the sections were incubated for 1 h with biotinylated secondary goat anti-rabbit IgG (Abcam, Cambridge, UK). After incubation, ABC Reagent (Vector Laboratories) was applied to react with the biotinylated antibody for 1 h at 25 °C and attached with the 3,3′-diaminobenzidine (DAB) reagent (Vector Laboratories). Mean staining intensity without DAB for syndecan-1 was standardized as a negative control. The slides were stained with haematoxylin as a counterstain, dehydrated, and cover-slipped using mounting medium (Vector Laboratories). Images were obtained through a microscope. Syndecan-1 intensity was quantified using ImageJ software.

### Endothelial damage according to blood syndecan-1 level

Syndecan-1 was measured to check damage to glycocalyx using an enzyme-linked immunosorbent assay (ELISA) (R&D Systems Inc., Minneapolis, MN, USA).

### Blood cytokine levels

Serum levels of interleukin (IL)-2, interferon (IFN)-γ, tumour necrosis factor (TNF)-α, and transforming growth factor (TGF)-β were determined using ELISAs (R&D Systems).

### Statistics

Previous report for the association between amount of fluid and immune response to determine sample size with power analysis has not been found. Therefore, sample size was determined using the resource equation method, instead of power analysis.

With the formula for the resource equation method (E = Total number of animals – Total number of groups, any sample size, which keeps E between 10 and 20, should be considered to be adequate.), total number of animals between 14 and 24 was adequate for sample size determination.

For intergroup comparisons, two-way repeated-measures analysis of variance (ANOVA) with the Bonferroni method as a post hoc test was used. For intragroup comparisons, one-way repeated-measures ANOVA with the Bonferroni method or Friedman’s test was used.

A *p*-value <0.05 was considered significant. Data are presented as mean ± standard deviation.

## Results

A total of 24 rats were evenly allocated into the four groups. All rats were successfully resuscitated with fluid therapy and were enrolled in the final analyses. The exsanguinated blood volumes were similar among the groups (7.79 ± 0.26 mL from 243.33 ± 8.16 g in the crystalloid-1 group [n = 6], 7.73 ± 0.37 mL from 241.67 ± 11.69 g in crystalloid-2 group [n = 6], 7.73 ± 0.24 mL from 241.67 ± 7.53 g in crystalloid-3 group [n = 6] and 7.73 ± 0.24 mL from 241.67 ± 7.53 g in the colloid group [n = 6], *p* = 0.984).

BP decreased significantly after exsanguination and was the lowest at T3 in all groups. BP was restored after fluid administration but BP at T6 was significantly lower than that at T0 in all groups. The changes in BP were similar in all groups (Table 2).

**Table 2:** The changes of blood pressure.

	Crystalloid-1 group	Crystalloid-2 group	Crystalloid-3 group	Colloid group	*p*
T0 (mmHg)	135.30 ± 6.59	133.40 ± 6.88	136.00 ± 7.07	132.30 ± 6.11	0.823
T1 (mmHg)	83.70 ± 12.65^*^	81.82 ± 13.45^*^	79.15 ± 9.78^*^	77.12 ± 12.56^*^	0.799
T2 (mmHg)	48.69 ± 8.99^*^	51.42 ± 4.37^*^	47.17 ± 6.35^*^	50.27 ± 6.69^*^	0.711
T3 (mmHg)	41.62 ± 7.41^*^	40.98 ± 4.82^*^	41.27 ± 5.63^*^	42.80 ± 8.85^*^	0.856
T4 (mmHg)	45.17 ± 9.73^*^	48.18 ± 4.65^*^	44.35 ± 5.54^*^	45.57 ± 6.34^*^	0.862
T5 (mmHg)	64.64 ± 7.60^*^	66.18 ± 5.20^*^	64.96 ± 8.59^*^	63.34 ± 5.04^*^	0.965
T6 (mmHg)	87.89 ± 5.67^*^	88.25 ± 7.86^*^	88.75 ± 5.94^*^	86.98 ± 12.02^*^	0.715

Data are expressed as mean ± standard deviation. **Abbreviations:** T0, before exsanguination; T1, at 10 minutes after exsanguination; T2, at 20 minutes after exsanguination; T3, at 30 minutes after exsanguination; T4, at 10 minutes after resuscitation; T5, at 20 minutes after resuscitation; T6, at 30 minutes after resuscitation. ^*^p < 0.05 compared to T0.

**Figure 1:**
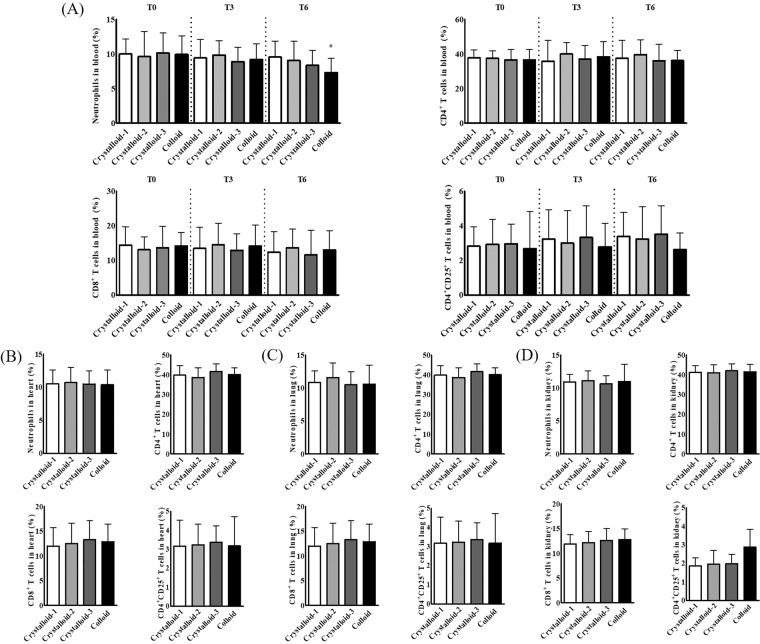
The expression of immune cells in the blood (**A**), heart (**B**), lungs (**C**), and kidneys (**D**) after resuscitation with different ratios of crystalloid or colloid. Abbreviations: CD, cluster of differentiation; T0, before exsanguination; T3, 30 min after exsanguination; T6, 30 min after resuscitation. ^*^p < 0.05 compared to T0.

The expression of immune cells in the blood, except neutrophils in the colloid group, did not change during exsanguination or fluid administration (Fig. 1). The expression of neutrophils in the blood decreased significantly after colloid administration, compared to before exsanguination (Fig. 1).

**Figure 2:**
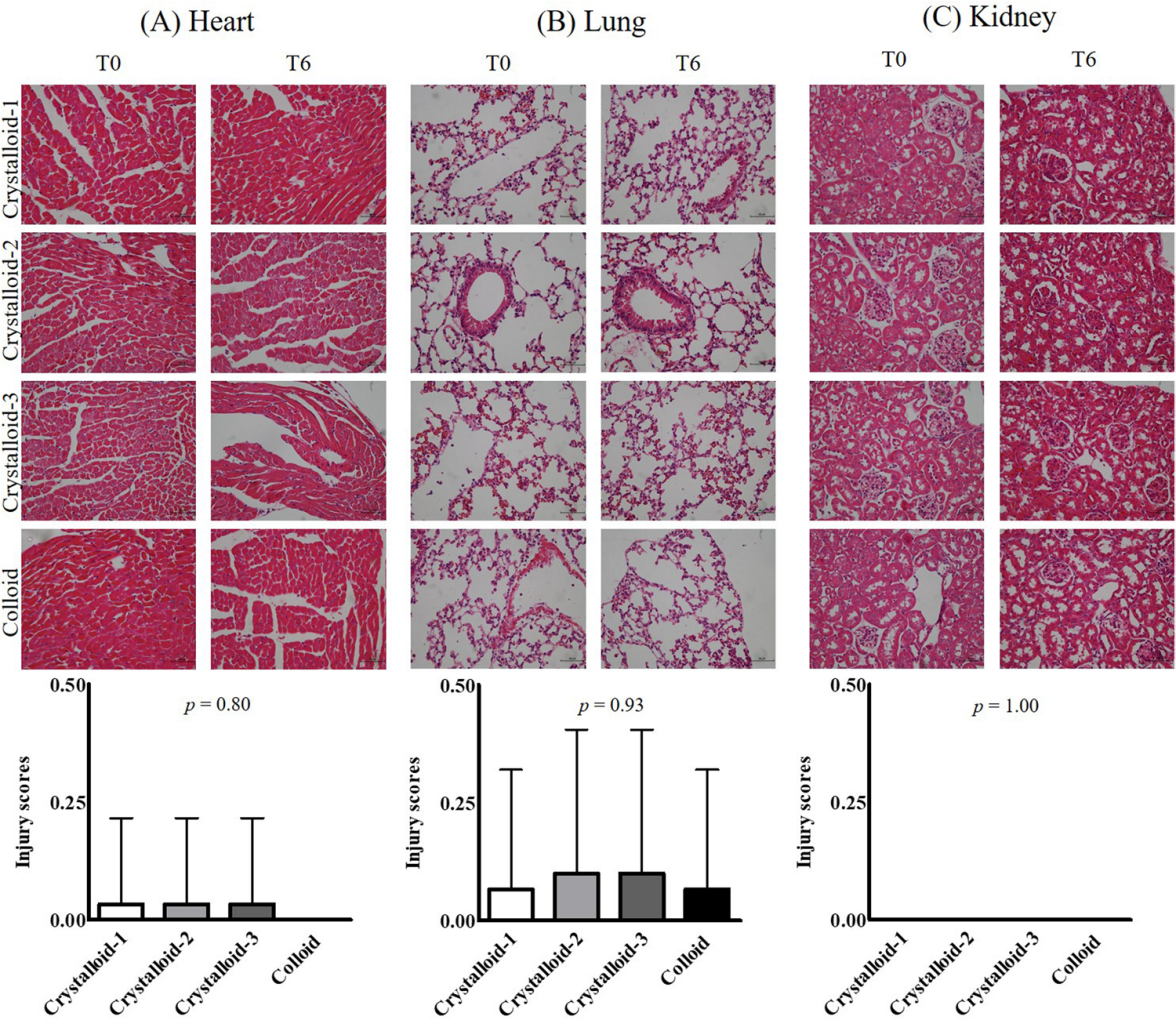
Histopathological analyses of the heart (**A)**, lungs (**B**), and kidneys (**C**) after resuscitation with different ratios of crystalloid or colloid.

**Figure 3:**
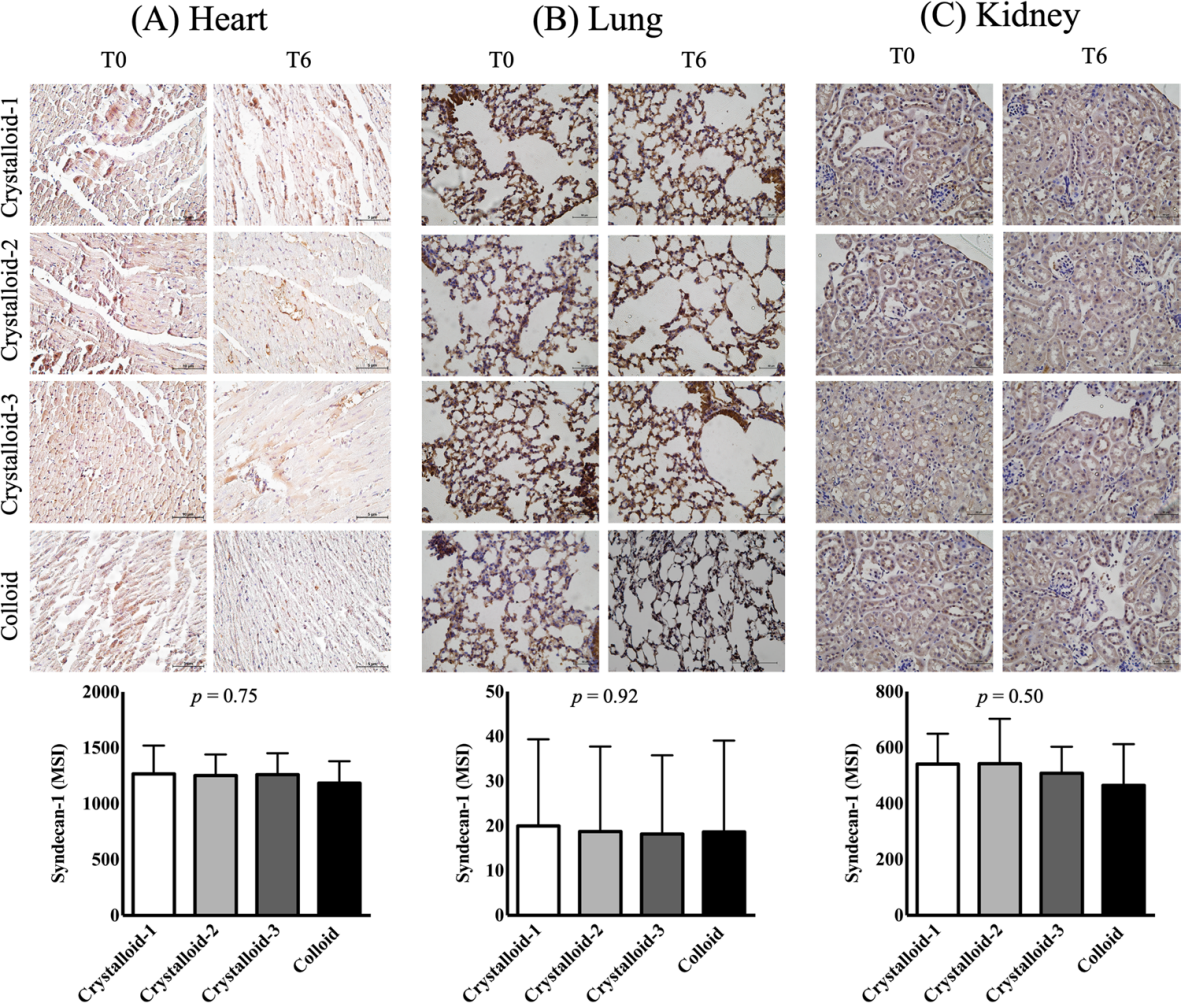
Immunohistochemical assay of the heart (**A**), lungs (**B**), and kidneys (**C**) after resuscitation with different ratios of crystalloid or colloid. Abbreviation: MSI, Mean Staining Intensity for syndecan-1.

**Figure 4:**
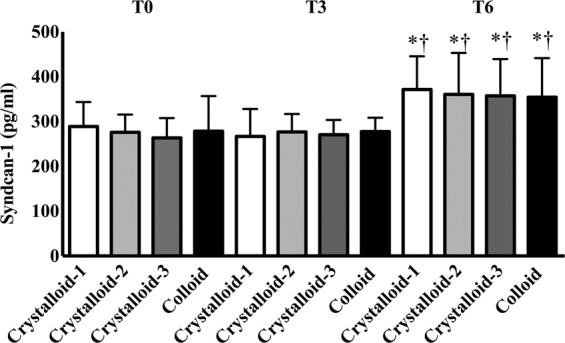
Endothelial damage according to blood levels of syndecan-1. Abbreviations: T0, before exsanguination; T3, 30 min after exsanguination; T6, 30 min after resuscitation. ^*^p < 0.05 compared to T0. ^†^*p* < 0.05 compared to T3.

In histopathological analyses, the injury scores for the heart, lungs, and kidneys were not significantly different among the groups (*p* = 0.80 in the heart, *p* = 0.93 in the lungs, and *p* = 1.00 in the kidneys) (Fig. 2). Syndecan-1 expression was not significantly different among the groups (*p* = 0.75 in the heart, *p* = 0.92 in the lungs, *p* = 0.50 in the kidneys) (Fig. 3). Blood syndecan-1 expression at T6 was significantly higher than that at T0 and T3 in all groups but no significant differences were observed among the groups (Fig. 4). Blood cytokine levels did not change during exsanguination or fluid administration. There were no differences among the groups (Table 3).

**Table 3:** The changes of cytokines in the blood.

	Crystalloid-1 group	Crystalloid-2 group	Crystalloid-3 group	Colloid group	*p*
**IL-2 (pg/ml)**					
T0	148.00 ± 5.93	144.00 ± 6.90	143.83 ± 7.78	137.00 ± 6.42	0.072
T3	144.00 ± 8.97	141.33 ± 9.61	143.33 ± 10.58	144.33 ± 6.50	0.940
T6	144.17 ± 10.93	144.00 ± 6.45	143.17 ± 7.08	142.33 ± 10.54	0.983
**IFN-γ (pg/ml)**					
T0	314.33 ± 12.45	312.67 ± 20.29	303.17 ± 16.19	317.67 ± 19.87	0.531
T3	310.67 ± 14.88	312.00 ± 19.29	313.17 ± 18.82	309.83 ± 14.39	0.987
T6	316.50 ± 27.70	308.50 ± 29.34	308.33 ± 22.66	320.17 ± 25.71	0.825
**TNF-α (pg/ml)**					
T0	14.50 ± 4.31	13.05 ± 3.78	11.67 ± 3.95	12.28 ± 3.04	0.609
T3	12.27 ± 3.84	12.50 ± 3.86	12.07 ± 3.98	11.63 ± 4.26	0.984
T6	12.77 ± 4.25	11.40 ± 3.87	12.23 ± 3.12	10.93 ± 3.30	0.823
**TGF-β (pg/ml)**					
T0	139.67 ± 11.48	144.00 ± 6.90	143.83 ± 7.78	137.00 ± 6.42	0.418
T3	134.67 ± 9.50	129.00 ± 9.94	129.50 ± 12.47	128.83 ± 10.05	0.741
T6	128.00 ± 7.75	127.33 ± 15.07	127.83 ± 9.93	127.00 ± 16.22	0.999

Data is expressed as mean ± standard deviation.

Abbreviations: IL, interleukin; IFN, interferon; TNF, tumor necrosis factor; TGF; TGF, transforming growth factor; T0, before exsanguination; T3, at 30 minutes after exsanguination; T6, at 30 minutes after resuscitation.

## Discussion

Fluid resuscitation using the same, two-fold, or three-fold volumes of balanced salt-based crystalloid or the same volume of balanced salt-based colloid resulted in a similar increase in BP in rats with haemorrhagic shock without any significant differences in syndecan-1 expression by immune cells. However, balanced salt-based colloid administration significantly reduced the expression of neutrophils in the blood.

The TEBV of rats is 5.8-7.0 mL/100 g^11^ and 30-60% of TEBV is usually shed for the shock model^12^. The model follows a fixed-volume haemorrhage. We removed 50% of TEBV to induce shock in the present study. Another popular model for haemorrhagic shock is the fixed-haemorrhage-pressure model, in which experimental animals are bled until BP reaches a predetermined level, at which point it is maintained. The target BP for haemorrhagic shock is 25-50 mmHg. The BP after exsanguination of all groups in the present study reached a similar level and was maintained. Although a fixed-volume haemorrhage was applied in the present study, the induced hypotensive BP was shown in the model for a fixed pressure haemorrhage and was associated with a similar age and body weight of the experimental animals. The duration of haemorrhagic shock, except BP, was the most crucial factor affecting the experimental animals. A duration of 15-180 min is applied for the mode^13,14^. BPs 20 min after exsanguination in all groups in the present study were ≤50 mmHg and were maintained until 20 min after resuscitation for approximate 60 min. Therefore, the haemorrhagic shock model in the present study was reasonable. Continuous BP monitoring, instead of intermittent BP monitoring, in the present study would be helpful to verify the adequacy of the model. Actually, no difference of BP restoration at different amount of fluid in the present study was remarkable. Considering Frank-Starling law, increased volume results in increased cardiac output, representing increased BP, until the peak is reached. After peak, additional volume deteriorates cardiac output. No difference of BP restoration at different amount of fluid might be interpreted as a similar volume status inside blood vessel, although volume status outside blood vessel might be different. Therefore, additional evaluation for micro-circulation would be more informative to determine the status after recovery from haemorrhagic shock. Moreover, BP in rat model for haemorrhagic shock was usually restored with the peak at just after fluid resuscitation and then gradually decreased due to ischaemia-reperfusion injury^15^. However, BP in the present study was gradually increased after fluid resuscitation. The different pattern of BP restoration might be associated with the degree of the tissue injury. Histopathologic analyses for the tissues from all groups in the present study showed less injury scores.

Glycocalyx is an intravascular barrier between the circulating blood and the vessel wall. It plays an important role in numerous physiological processes, including regulation of vascular permeability, prevention of marginated blood cells to the vessel wall, and transmission of shear stress^16^. A haemorrhage is the result of damage to the endothelial glycocalyx. Fluid resuscitation can also change the endothelial glycocalyx. We checked syndecan-1 level as an index of endothelial glycocalyx degradation in the present study. Haemorrhagic shock induces shedding of syndecan-1, which is associated with organ damage^17,18^. Resuscitation with plasma, not fluid, reduces shedding of syndecan-1 and reconstitutes the endothelial glycocalyx^17,19^. In the present study, the expression of syndecan-1 in the blood of all groups increased after exsanguination and fluid administration. However, the increase in syndecan-1 expression was not different among the groups. We simply expected that the colloid group would have a different syndecan-1 expression level compared to the crystalloid groups because HES sometimes has a role of an allergen and that the crystalloid-1 group would show less expression compared to the crystalloid-3 group because lesser volume amount would less deteriorate glycocalyx. The lack of a significant difference in syndecan-1 levels might be explained as follows. László I *et al*. reported that volume replacement ratios for haemodynamic restoration were 0.92 with colloid and 3.03 with crystalloid, respectively, in moderate bleeding animal, but there was no significant difference for glycocalyx degradation between the groups^20^. Torres *et al*. reported that 75 mL/kg (above three fold for blood loss) lactated Ringer’s solution and 15 mL/kg hetastarch restores haemodynamic parameters with similar expression of syndecan-1 levels in the sera of rats in haemorrhagic shock^21^. Kim *et al*. reported that plasmalyte and 6% hetastarch results in similar microvascular reactivity with degradation of endothelial glycocalyx in patients undergoing off-pump coronary artery bypass graft surgery, although the plasmalyte was significantly more infused (less two-fold)^22^. The fluids used in above the two studies of Torres *et al*. and Kim *et al*. were all balanced salt-based crystalloid and colloid. Resuscitation with normal saline based solution results in a significantly higher expression of plasma syndecan-1 compared to that after administration of balanced salt-based solution^23^. The type of crystalloid and colloid in the present study was a balanced salt-based solution, not normal saline. Therefore, the expression of syndecan-1 in the blood was not significantly different among the groups. However, the interpretation for syndecan-1 would need the further evaluation. As we mentioned above, regarding no difference of BP restoration at different amount of fluid, different volume status outside blood vessel might impact the expression of syndecan-1 in the blood as time goes.

The increased expression of syndecan-1 from degradation of the endothelial glycocalyx during haemorrhagic shock stimulates immune cells, which then produce cytokines^17^. The similar syndecan-1 expression levels among the groups may be associated with the lack of a difference in cytokine expression by immune cells. This would also result in a lack of difference in the histopathological analyses and immunohistochemistry assay in the tissues among the groups.

The experimental animals in the present study were sacrificed 60 min after fluid administration. The results under a longer observation period might be different. In fact, we expected that administering different ratios of crystalloid or colloid would result in differences in BP restoration, which would influence the immune response. Therefore, we thought that a limited observation period would be helpful to determine the pure effects of administering different ratios of crystalloid or colloid on the immune response, ruling out the effects of BP. However, we found no differences.

Colloid reduces the expression of neutrophils^24–26^. In a previous study, we co-cultured mouse splenocytes with colloid, containing HES, and crystalloid which were same colloid and crystalloid in the present study. We found that colloid, containing HES, reduces the expression of neutrophils compared to crystalloid^27^. In the present study, the expression of neutrophils was significantly lower in the colloid group than in the crystalloid groups. However, it did not affect the immune response to tissue injury.

Cytokine levels did not increase after haemorrhagic shock and resuscitation, although the level of syndecan-1 in the blood increased. However, similar expression patterns of syndecan-1 were detected in the groups, which resulted in the lack of a difference in the expression of cytokines among the groups.

Several limitations should be considered in the study. First, we did not check arterial blood gas analysis (ABGA) with electrolytes. The changes, including acid-base balance, before and after fluid administration would give more information. However, the values might not have any significant difference between the groups due to similar composition of the crystalloid and the colloid in the present study. Second, we did not measure any functional data to check organ injury, including an indicator for shock such as lactate level. The data would be useful. However, the changes for the values before and after haemorrhage with resuscitation would be not be special with no differences in histopathological analyses for the limited observation in the present study.

In conclusion, same volume of balanced salt-based crystalloid for blood loss was enough to restore BP at the choice of fluid for the management of haemorrhagic shock in the rats, compared with different ratios of crystalloid or same volume of colloid, on the aspect of immune response.
